# The Effectiveness of an Educational Intervention on Knowledge, Attitudes and Reported Practices on Antibiotic Use in Humans and Pigs: A Quasi-Experimental Study in Twelve Villages in Shandong Province, China

**DOI:** 10.3390/ijerph18041940

**Published:** 2021-02-17

**Authors:** Liyan Shen, Oliver James Dyar, Qiang Sun, Xiaolin Wei, Ding Yang, Chengtao Sun, Yang Wang, Hongyu Li, Yuqing Liu, Yanbo Luo, Jia Yin, Cecilia Stålsby Lundborg

**Affiliations:** 1 NHC Key Lab of Health Economics and Policy Research, Centre for Health Management and Policy Research, School of Public Health, Cheeloo College of Medicine, Shandong University, Jinan 250012, China; 17862957443@163.com (L.S.); qiangs@sdu.edu.cn (Q.S.); yangding@sdu.edu.cn (D.Y.); lhydevil@163.com (H.L.); 2 Department of Global Public Health, Karolinska Institutet, 17177 Stockholm, Sweden; oliver.dyar@ki.se (O.J.D.); cecilia.stalsby.lundborg@ki.se (C.S.L.); 3 Dalla Lana School of Public Health, University of Toronto, Toronto, ON M5S 2E8, Canada; xiaolin.wei@utoronto.ca; 4 Beijing Advanced Innovation Center for Food Nutrition and Human Health, College of Veterinary Medicine, China Agricultural University, Beijing 100083, China; sunctx@icloud.com (C.S.); vetwangyang@163.com (Y.W.); 5 Institute of Animal Science and Veterinary Medicine, Shandong Academy of Agricultural Science, Jinan 250100, China; liuiuqing@163.com (Y.L.); luoyanbosaas@163.com (Y.L.)

**Keywords:** antibiotic, educational intervention, humans and animals, China

## Abstract

Our aim was to evaluate the effectiveness of an intervention for residents in rural China on knowledge, attitudes and reported practices (KAP) on antibiotic use in humans and pigs. A quasi-experimental study was conducted in 12 villages in rural Shandong province, divided into intervention and control groups, covering a two-year period from July 2015 to June 2017. A package of health education-based interventions including training sessions, speakerphone messages, posters and handbooks for residents was developed and implemented over a one-year period to improve the use of antibiotics in humans and pigs. The intervention net effects were evaluated by Difference-in-Difference (DID) analysis based on responses to a questionnaire concerning KAP towards antibiotic use in humans and pigs. A total of 629 participants completed both baseline and post-trial questionnaires, including 127 participants with backyard pig farms. Significant improvements were found in KAP towards antibiotic use in humans, but changes related to antibiotic use for pigs were not significant. Participants who were in the intervention group (*p* < 0.001) were more likely to have improved their knowledge on antibiotic use in humans. Participants who had higher attitude scores were less likely to report self-medicating with stored antibiotics in the previous year (*p* < 0.001). Our results suggest that our health education-based intervention was effective in improving KAP on human antibiotic use, but it had little effect regarding antibiotic use for pigs.

## 1. Introduction

The emergence and spread of bacterial resistance to antibiotics is a growing problem worldwide [1]. Over-use and misuse of antibiotics in animals and humans are contributing to the rising problems of resistance, and the “One Health” framework has been recommended by the WHO to govern the intersectoral issue of antibiotic resistance [1,2,3]. China is one of the largest consumers of antibiotics globally, over half of which are used on animals [4,5].

In Shandong province, over 70% of the total antibiotic consumption in public healthcare institutions were used in rural primary care facilities [6]. Moreover, inappropriate use of antibiotics for humans and animals has commonly been reported by studies conducted in rural areas [7,8]. Among patients, low levels of education and serious misconceptions regarding antibiotics have been identified as important factors related to antibiotic overuse [9]. Most efforts to improve the situation, however, have focused on providers [10,11]. Training only providers may have a limited impact on the rational use of antibiotics in rural primary care settings because of the financial incentives from selling medicines and perceived patient demand [3,4].

Several studies have shown that educational interventions can enhance public knowledge about appropriate use of antibiotics and resistance to antibiotics, and that they can be relatively resource-efficient methods. Interventions have included training sessions, educational pamphlets, posters, and media interventions such as television and radio on appropriate antibiotic use [12,13,14]. However, compared with high income countries such as the United Kingdom and Sweden [15,16], China is lagging behind on educational efforts to improve antibiotic-related knowledge for consumers. Up to now, few studies have used multifaceted interventions to promote rational use of antibiotics for humans, and even less for use of antibiotics in animals [17]. We developed a pilot package of One Health interventions and assessed their effect on rural residents’ knowledge, attitudes and reported practices (KAP) towards antibiotic use in humans and animals, as part of the Sino-Swedish Integrated Multisectoral Partnership for Antibiotic Resistance Containment (IMPACT) [18].

## 2. Methods

### 2.1. Study Design and Setting

From July 2015 to June 2017, a two-year quasi-experimental study was conducted in a county in Shandong, a province in which 7% of the Chinese population live [19]. The rural areas of Shandong are generally similar to other rural areas in eastern China in terms of education, health indicators, and per-capita income.

The full study protocol for the IMPACT research program has previously been published [18]. Participating households were selected using a multistage cluster sampling method in an area in a county, using background data collected by the local Centers for Disease Control and Prevention. A power calculation [18] was used to determine the number of households needed for all components of the IMPACT research program. Firstly, 12 of 17 villages with >100 households in each village were selected to maximize: (i) the number of included backyard pig farms; and (ii) villages that had human healthcare clinics. Then, 65 households in each of these 12 villages were selected to produce a total of 780 households. The 12 villages were subsequently purposefully divided into six control villages and six intervention villages, by considering the number of households with and without pigs in each village, and the location of shared village healthcare clinics. This was to ensure that a similar number of households with and without pigs were included in the intervention and control groups, and to ensure that village clinics were not shared between intervention and control groups.

### 2.2. Participants

Households as a whole participated in the intervention, but only one member of each participating household completed the baseline and post-trial questionnaire. Participation in the study was voluntary. A gift worth 20 yuan (approximately €2) was given as an incentive to each participating household that responded to the questionnaire or attended a training session. 

### 2.3. Intervention

A package of visual and auditory intervention materials was developed, based on the literature review and the findings of the baseline survey [19], aiming to improve the KAP of rural residents on antibiotic use in humans and on backyard pig farms. A panel of experts in clinical medicine, microbiology, veterinary medicine, health policy and management helped to refine the intervention package. We implemented the intervention package for a period of one year (July 2016-June 2017). The intervention included the following four components (for details, see Supplementary Materials):

1. Training sessions: All the participants of each village were invited to participate in a two-hour training session on antibiotic use in every quarter (August and November 2016, March and June 2017), at the village’s community center. Two chief physicians from the county hospital serving the area provided the training. They received instructions on the purposes and an outline of the training at the beginning from the project coordinator of Shandong University. Photos and videos regarding the harms of irrational use of antibiotics were used as Supplementary Materials during the training sessions. The sessions were interactive and participants were encouraged to ask questions.
2. Speakerphone: Traditionally, there is a speakerphone hung on a pillar in each village in China, which is used to notify villagers of important affairs. The sounds they produce can be heard throughout the entire village. We drafted and recorded three dialogues between two voice actors. One actor played a village resident who asked questions regarding antibiotic use for humans and pigs, and another one played a doctor and veterinarian who answered these questions. In each intervention village, a social worker was employed to broadcast the 15-min dialogues once in the morning and once in the evening, every Tuesday, Thursday and Saturday for a period of one year.
3. Posters: In each intervention village, four posters were posted at gathering places, such as the door of a retail store, the wall of a village clinic, or a bulletin board. The posters contained several simple and easily understandable cartoons to show how to use antibiotics rationally. Every quarter, the posters were replaced by new ones with identical content.
4. Handbook: An educational handbook was developed based on reviews of relevant international and national guidelines and comments from experts in clinical medicine, public health, animal health and health policy. It detailed basic knowledge about antibiotics and how they should and should not be used. The handbook was distributed to all the households in the intervention villages in August 2016 after the first training session.

### 2.4. Data Collection

#### 2.4.1. Baseline and Repeated Survey

A household questionnaire was developed before baseline data collection based on the literature review, expert consultation, pilot study, and our previous work [20]. The questionnaire consisted of participants’ socio-demographic characteristics, and their KAP concerning antibiotic use for humans and pigs. The baseline and post-trial data collection was conducted in July 2015 and July 2017, respectively. Each participating household was visited and an adult member over the age of 18 was invited to respond orally to the questionnaire. The individual who responded to the baseline questionnaire was requested to respond to the post-trial questionnaire. The data collectors were master’s students from local universities, and they had attended a half-day of training on how to conduct questionnaires. 

#### 2.4.2. Process Evaluation

To evaluate the experiences and perceived quality of the intervention components, we conducted two process evaluations in March and June 2017, respectively. In each process evaluation, around 15 residents per intervention village from different households who participated in the intervention were invited to participate. They were asked questions regarding their participation in and satisfaction with the different intervention components. Participants were firstly asked about the accessibility of each intervention with an answer of “yes” or “no”. Then, the satisfaction of each intervention was investigated using four questions regarding its acceptability, understandability, and perceived effectiveness on improving knowledge and practices. The selected participants could give a score to each question on a scale from 0 to 5. 

### 2.5. Data Analysis

All the data were double-entered in Microsoft Access 2007(Microsoft Corporation, Raymond, WA, USA). We matched the data collected from baseline and repeated surveys according to household ID. SPSS (version 22.0, IBM Corporation, Armonk, NY, USA) and STATA (version 14, Stata Corp, Texas, TX, USA) were used to analyze data. 

Difference-in-Difference (DID) analysis was employed to identify the changes of KAP towards antibiotic use for each individual pre- and post-trial in the two arms. Multiple linear regressions (MLRs) were used to identify factors associated with changes in KAP. For each question, points were assigned to the pre-and post-trial answers for each resident. If the resident changed their answer from inappropriate to appropriate (i.e., a KAP item likely associated with inappropriate antibiotic use, to one associated with appropriate antibiotic use), he/she would get four points (progressive). If the answer was appropriate both times, they would be given three points (positively stable), and if both answers were inappropriate, two points would be given (negatively stable). If the change was from appropriate to inappropriate, they would get one point (retrogressive). Then, the total score of knowledge and attitudes were summed up by each question for each resident, and were taken as a dependent variable in the MLRs, and independent variable in the logistic regressions. Independent variables also included variables of intervention, gender, age, educational level, and per capita income. Two reported practices were treated as dependent variables in the logistic regressions. Due to the small sample size, factors associated with KAP on antibiotic use in pigs were not analyzed. Statistical significance was set at *p* < 0.05 for all comparisons. 

## 3. Result

### 3.1. Characteristics of Respondents

At baseline, a total of 389 and 380 rural residents were recruited into intervention and control groups, respectively. In total, 140 participants were lost to follow-up for reasons including out-migrating for work (67/140, 47.9%), moving out (40/140, 28.6%), suffering from serious illnesses (12/140, 8.6%), death (12/140, 8.6%), and refusal (9/140, 6.4%). After matching the baseline and post-trial, 321 and 308 respondents in the intervention and control groups were included for analysis. Among these respondents, 220 (baseline) and 199 (post-trial) residents had backyard pig farms at the time of surveys; of these, 127 (intervention: 59; control: 68) respondents had backyard pig farms at the time of both surveys. Compared with the respondents in the control group, those in the intervention group had higher level of education (*p* = 0.015) and higher per capita income (*p* < 0.001) (Table 1).

### 3.2. Changes in Knowledge, Attitudes and Reported Practices Pre- and Post-Trial

After adjusting for potential confounders, responses to each of the questions related to knowledge on antibiotic use in humans was found to have improved to a greater extent in the intervention group than the control group at the post-trial. Similarly, attitudes towards antibiotic use in humans improved more in the intervention group than in the control group, for all but one item. There were also greater improvements in the intervention group with response to self-reported practices towards antibiotic use in humans (Table 2). Overall, there were no major changes in the KAP towards antibiotic use in pigs when comparing intervention and control groups (Table 3).

### 3.3. The Association between KAP on Antibiotic Use in Humans 

The results of the MLR analysis indicated that participants who were in the intervention group (*p* < 0.001) were more likely to have higher scores of knowledge on antibiotic use in humans. Participants who had higher knowledge scores (*p* < 0.001) with a higher level of education (*p* = 0.012) and those who were in the intervention group (*p* < 0.001) tended to have higher scores on attitudes. Participants who had higher attitude scores were less likely to report having self-medicated with stored antibiotics in the previous year (*p* < 0.001) (Table 4).

### 3.4. Process Evaluation

A total of 184 residents in the intervention group were interviewed during the process evaluations. The majority of the respondents had attended a training session (99.5%) and had heard the speakerphone broadcasts (84.8%), whereas less than one third of the respondents had read the posters (27.7%) or educational handbook (32.1%) (Table 5). Almost all of the respondents thought that the time arrangement and structure of the intervention were acceptable. More than three-quarters of the respondents thought that the contents of the training session and the speakerphone broadcasts were easy to understand. Most respondents believed that the different intervention materials could improve their knowledge and practices on antibiotic use.

## 4. Discussion

Based on the One Health approach, we designed a multifaceted educational package. The results showed that the intervention package was effective in improving rural residents’ knowledge and attitudes towards rational antibiotic use in humans, which was consistent with the findings reported by previous studies implemented educational intervention on health providers [3,12,14]. However, there was no significant effect of our intervention on improving KAP of antibiotic use in pigs.

In order to evaluate the experiences and perceived quality of different types of interventions, we conducted two process evaluations during the period of the intervention. These showed that a higher proportion of participants accessed the auditory interventions than the visual interventions. Written materials like posters and handbooks are more suitable for people who are capable of reading. In China, rural-to-urban migration, particularly of younger adults, has led to an aging rural population. These older people often have medical problems of presbyopia as well as low literacy levels. This may explain why only a small proportion of participants reported accessing the posters and handbooks in this study. Among the auditory interventions, the speakerphone was confirmed to be more effective than verbal training on improving residents’ knowledge [21]. In rural China, the speakerphone is an essential item in each village and is often used for notifying messages and entertainment using dialects. It requires no prior standard of education and would not disturb the routine work of villagers. Because of the convenience and cost-effectiveness, the speakerphone is highly recommended in improving the knowledge level of general population in low-and middle-income countries (LMICs) where such speakerphones are available [22].

Residents in our study appeared to have low knowledge on antibiotic use and resistance compared with residents in other countries (46–73% on average) [23]. Even after intervention, the participants still lack knowledge on antibiotic resistance. The concept of resistance of bacteria to antibiotics is quite abstract and can be difficult to understand for those who have no medical background [13]. For general populations with a low level of education, understandable knowledge focus on antibiotic resistance should be given in the future, such as an emphasis on positive messages that empower individuals (e.g., use of symptomatic treatment and hand washing) is likely to be more constructive. No significant difference was found after intervention regarding the attitude of taking antibiotics for a full course by following doctor’s advice [24]. One explanation was that the consumers, especially those living in lower-income areas, thought it may create unnecessary expenses if they kept taking medicine after they started feeling better. For this reason, they may have preferred to keep leftover antibiotics from the incomplete course of treatment for future self-medication [25]. Another reason, as reported by a study conducted in rural China [26], was that village doctors often do not explain a condition or treatment regimen to patients. Based on this, it is also vital to train the village doctors to educate patients on the correct use of prescribed antibiotics.

For most of the questions of KAP regarding antibiotic use for pigs, the appropriate answer rates increased in both groups. Thus, the differences between the two groups before and after intervention were not significant. In mid-2016 and early-2017, the government of Shandong Province launched provincial campaigns to curb overuse of antibiotics in livestock and poultry and control the environmental pollution caused by feces of livestock and poultry, respectively. One of the actions was to introduce the harm of irrational antibiotic use for animals to rural residents. These policies could have contributed to the improvement in both groups. However, it might also indicate that our intervention did not have extra effectiveness on improving KAP on antibiotic use in pigs. Some farmers reported using antibiotics to keep their pigs healthy, so that they would not lose money. Previous studies have suggested that farmers perceive income as a much more important issue than health [27,28]. In addition, education on antibiotic use for animals was not included in all components our of intervention nor was it strongly focused on pig farmers in this study.

An important strength of our study design is that we were able to conduct our intervention over a one-year period with repeated speakerphone messages, quarterly training sessions and quarterly renewal of posters. Using repeated efforts likely strengthened the impact of the individual intervention components on residents’ KAP, as well as how sustained their impact will be in the long-term, as has been suggested in recent systematic reviews of interventions to improve antibiotic use both in community and hospital settings [29,30]. If the intervention is to achieve permanent effects, we think it is likely to need further support from the local government, for example through continued, but less frequent, use of the speakerphone and training sessions.

Our study has additional strengths. Firstly, it designed and implemented interventions on the use of both human and animal antibiotics based on a “One Health” framework. Secondly, we conducted two process evaluations during the intervention period, which helped to evaluate the performance of different types of interventions. Thirdly, we developed a package of visual and auditory interventions, among which, the speakerphone was confirmed to be a feasible and accessible intervention that could be used in low-resource settings. In order to work towards the long-term sustainability of the intervention measures, we have subsequently submitted a policy brief report to the local and provincial government, suggesting that the speakerphone continue to be used to broadcast health-education messages both in the study villages and beyond.

This study also has several limitations. Firstly, it was conducted in a single county due to time and funding limitation. However, this county is representative of rural China in terms of its education, health indicators, and per-capita income. Secondly, only half of the farmers with backyard pigs at baseline survey still raised pigs at post-trial. Thus, the number of participants who responded to the animal questionnaire both before and after intervention was small (*n* = 127), and the effectiveness of our interventions on antibiotic use for pigs could not be fully evaluated. Thirdly, we conducted our post-trial survey one-month after the final training session, so we are unable to provide data on the longer-term impact of our intervention on residents’ KAP. Finally, there are other potential confounding factors that it was not possible to account for in our statistical analyses (such as recent medical exposures, underlying health status of the respondents), although we have no strong reasons to believe that these would have differed between control and intervention villages.

## 5. Conclusions

Our results show that a package of visual and auditory educational intervention was effective in improving the KAP on antibiotic use in humans in rural residents. However, the intervention had little effect on the KAP of backyard pig farmers in terms of rational use of antibiotics for their pigs. Among the four types of intervention, the speakerphone was considered the most feasible and accessible, and could be generalized to other LMICs with similar conditions of inappropriate use of antibiotics.

## Figures and Tables

**Table 1:** Socio-demographic characteristics of the participants.

Characteristics	All Participants			Participants with Backyard Pig Farms		
	Intervention (N = 321), No. (%)	Control (N = 308), No. (%)	*p*-Value	Intervention (N = 59), No. (%)	Control (N = 68), No. (%)	*p*-Value
Gender						
Male	177 (55.1)	191 (62.0)	0.080	41 (69.5)	45 (66.2)	0.690
Female	144 (44.9)	117 (38.0)		18 (30.5)	23 (33.8)	
Age, median (IQR)	53 (48, 61)	54 (48, 64)	0.017	55 (50, 61)	54 (50, 60)	0.975
Educational level						
Primary school or below	180 (56.1)	202 (65.6)	0.015	27 (45.8)	40 (58.8)	0.141
Middle school or above	141 (43.9)	106 (34.4)		32 (54.2)	28 (41.2)	
Per capita income, median (IQR)	6667 (3333, 10,000)	5000 (2000, 10,000)	<0.001	10,000 (3333, 16,667)	10,575 (4250, 17,500)	0.651

No.: Number of respondents. IQR: Interquartile Range.

**Table 2:** Effect of intervention on knowledge, attitudes and reported practices on antibiotic use in humans.

Questions	Answer	Intervention (N = 321)			Control (N = 308)			*p* Value
		Baseline, No. (%)	Post-Trial, No. (%)	Δ_(0–1)_ *, No. (%)	Baseline, No. (%)	Post-Trial, No. (%)	Δ_(0–1)_ *, No. (%)	
Knowledge								
Participants correctly identified three antibiotics from a list of twelve medicines **	-	33 (10.3)	172 (53.6)	152 (47.4)	43 (14.0)	73 (23.7)	48 (15.6)	<0.001
Antibiotics should always be used whenever an adult has a fever	No	88 (27.4)	252 (78.5)	181 (56.4)	93 (30.2)	101 (32.8)	62 (20.1)	<0.001
Antibiotics should always be used whenever a child has a fever	No	93 (29.0)	249 (77.6)	175 (54.5)	90 (29.2)	99 (32.1)	54 (17.5)	<0.001
Antibiotics can be taken in advance to protect from the common cold	No	95 (29.6)	255 (79.4)	178 (55.5)	98 (31.8)	126 (40.9)	74 (24.0)	<0.001
Antibiotics can prevent the common cold from developing into more severe diseases, such as pneumonia	No	49 (15.3)	138 (43.0)	114 (35.5)	39 (12.7)	29 (9.4)	20 (6.5)	<0.001
Bacteria can become resistant to antibiotics	Yes	77 (24.0)	127 (39.6)	85 (26.5)	83 (26.9)	45 (14.6)	15 (4.9)	<0.001
Bacteria that are resistant to antibiotics can infect you or your family	Yes	74 (23.1)	111 (34.6)	76 (23.7)	78 (25.3)	33 (10.7)	18 (5.8)	<0.001
Total Score, median (IQR) ***	-	-	-	21 (18, 24)	-	-	16 (15, 18)	<0.001
Attitudes								
I should not ask the doctor to prescribe me antibiotics when I feel it is needed.	Agree	108 (33.6)	228 (71.0)	147 (45.8)	96 (31.2)	173 (56.2)	111 (36.0)	0.018
I believe that antibiotics should only be purchased with a prescription from a doctor	Agree	126 (39.3)	225 (70.1)	138 (43.0)	131 (42.5)	179 (58.1)	84 (27.3)	0.003
When taking antibiotics, even if I start to feel better, I should take the full course based on the doctor’s advice.	Agree	108 (33.6)	94 (29.3)	60 (18.7)	101 (32.8)	83 (26.9)	50 (16.2)	0.748
I am worried about antibiotic resistance	Agree	80 (24.9)	182 (56.7)	133 (41.4)	72 (23.4)	89 (28.9)	50 (16.2)	<0.001
I believe that my own practices towards controlling antibiotics resistance are important	Agree	53 (16.5)	170 (53.0)	137 (42.7)	67 (21.8)	82 (26.6)	50 (16.2)	<0.001
Total Score, median (IQR) **	-	-	-	14 (12, 16)	-	-	12 (11, 14)	<0.001
Reported practices								
Has bought antibiotics from the pharmacy in the past twelve months	Yes	113(35.2)	53(16.5)	-	80(26.0)	72(23.4)	-	-
Has bought antibiotics from the pharmacy without a prescription in the past twelve months	Yes	49 (15.3)	24 (7.5)	46(14.3)	39 (12.7)	34(11.0)	30(9.7)	0.043
Has self-medicated with stored antibiotics in the previous year	No	152 (47.4)	243 (75.7)	127 (39.6)	181 (58.8)	206 (66.9)	69 (22.4)	<0.001

DID analysis was used with adjustments made for age, education level and per capital income. No.: Number of respondents. * The number and percentage of participants whose answers changed from inappropriate to appropriate. ** Participants were shown photos and names of 12 commonly used medicines, of which 4 were antibiotics. Then, they were asked to pick three medicines that were antibiotics from the 12 medicines. *** Independent samples T test was used.

**Table 3:** Effect of intervention on knowledge, attitudes and reported practices on antibiotic use in pigs.

Questions	Answer	Intervention (N = 59)			Control (N = 68)			*p*-Value
		Baseline, No. (%)	Post-Trial, No. (%)	Δ_(0–1)_ * No. (%)	Baseline, No. (%)	Post-Trial, No. (%)	Δ_(0–1)_ * No. (%)	
Knowledge								
Antibiotics should be used whenever a pig stops eating its feed	No	24 (40.7)	35 (59.3)	19 (32.2)	27 (39.7)	21 (30.9)	13 (19.1)	0.025
Antibiotics should be kept left over at the farm to use again by self-experience	No	16 (27.1)	22 (37.3)	15 (25.4)	17 (25.0)	29 (42.7)	19 (27.9)	0.507
Attitudes								
I know when my pigs need medications	Agree	46 (78.0)	41 (69.5)	9 (15.3)	53 (77.94)	49 (72.06)	11 (16.2)	0.832
I would trust veterinarians if they decided to give a medication to a pig with an infection	Agree	13 (22.00)	51 (86.4)	39 (66.1)	17 (25.0)	60 (88.24)	45 (66.2)	0.885
Reported practices								
Used antibiotics only for pigs suffering from diseases	Yes	37 (62.7)	43 (72.9)	15 (25.4)	27 (39.7)	46 (67.7)	30 (44.1)	0.275
Has purchased antibiotics for pigs in the past year without asking for advice from a vet.	No	33 (55.9)	44 (74.6)	14 (23.7)	43 (63.2)	52 (76.5)	19 (27.9)	0.733

No.: Number of respondents. DID analysis was used with adjustments made for age, education level and per capital income. * The number and percentage of participants whose answers changed from inappropriate to appropriate.

**Table 4:** Multiple linear regression analysis for improvement on antibiotic use for humans.

Dependent Variables	Independent Variables	β (95% CI)	*p*-Value
Total score of knowledge	Constant	16.48 (15.08,17.89)	<0.001
	Intervention (0 = no; 1 = yes)	4.59 (4.04, 5.14)	<0.001
	Gender (0 = female; 1 = male)	0.06 (−0.54, 0.66)	0.852
	Age (median = 56, 0 ≤ 56; 1 > 56)	−0.24 (−0.83, 0.36)	0.428
	Educational level (0 = primary school or below; 1 = middle school or above)	0.14(−0.50, 0.77)	0.675
	Per capita income (median = 5500, 0 ≤ 5500; 1 > 5500)	−0.03 (−0.61, 0.55)	0.922
Total score of attitudes	Constant	10.57 (9.01, 12.13)	<0.001
	Intervention (0 = no; 1 = yes)	1.25 (0.71, 1.79)	<0.001
	Gender (0 = female; 1 = male)	0.07 (−0.42, 0.56)	0.767
	Age (median = 56, 0 ≤ 56; 1 > 56)	0.002 (−0.48, 0.49)	0.993
	Educational level (0 = primary school or below; 1 = middle school or above)	0.66 (0.15, 1.18)	0.012
	Per capita income (median = 5500, 0 ≤ 5500; 1 > 5500))	0.21 (−0.26, 0.68)	0.38
	Total score of knowledge	0.08 (0.01, 0.14)	0.016
Has bought antibiotics from the pharmacy without a prescription (0 = Yes, 1 = No)	Intervention (0 = no; 1 = yes)	0.45 (−0.25, 1.15)	0.207
	Gender (0 = female; 1 = male)	−0.35 (−1.00, 0.30)	0.291
	Age (median = 56, 0 ≤ 56; 1 > 56)	0.76 (0.11, 1.41)	0.022
	Educational level (0 = primary school or below; 1 = middle school or above)	−0.85 (−1.49, −0.21)	0.01
	Per capita income (median = 5500, 0 ≤ 5500; 1 > 5500))	0.14 (−0.45, 0.74)	0.636
	Total score of knowledge	−0.01 (−0.10, 0.07)	0.805
	Total score of attitudes	0.10 (−0.01, 0.20)	0.062
Reported practice of not self-medicating with stored antibiotics (0 = No, 1 = Yes)	Intervention (0 = no; 1 = yes)	0.32 (−0.10, 0.73)	0.133
	Gender (0 = female; 1 = male)	0.34 (−0.04, 0.72)	0.077
	Age (median = 56, 0 ≤ 56; 1 > 56)	0.13 (−0.24, 0.51)	0.484
	Educational level (0 = primary school or below; 1 = middle school or above)	−0.31 (−0.72, 0.09)	0.125
	Per capita income (median = 5500,0 ≤ 5500; 1 > 5500))	0.01(−0.35, 0.37)	0.957
	Total score of knowledge	0.02 (−0.03, 0.07)	0.388
	Total score of attitudes	0.16 (0.10, 0.26)	<0.001

β: Non-standardized beta coefficient. CI: Confidence interval.

**Table 5:** Process evaluation on the performance of four types of intervention.

Satisfaction with the Different Intervention Components	Training Session, No. (%) *	Speakerphone, No. (%) *	Posters, No. (%) *	Booklets, No. (%) *
I have heard or read in this type of education	183 (99.5)	156 (84.8)	51 (27.7)	59 (32.1)
The time arrangement and structure of this type of education are reasonable. (acceptability)	180 (98.4)	146 (93.6)	50 (98.0)	58 (98.3)
The content of this type of education is easy to understand. (understandability)	145 (79.2)	120 (76.9)	45 (88.2)	53 (89.8)
My awareness of the rational use of antibiotics has been improved due to this type of education. (effectiveness on knowledge)	138 (75.4)	115 (73.7)	41 (80.4)	46 (78.0)
This type of education has improved how I use antibiotics. (effectiveness on reported practice)	126 (68.9)	106 (73.9)	38 (74.5)	42 (71.2)

No.: Number of respondents. * The number and percentage of participants whose score of answer is from 3 to 5 for each question.

## Data Availability

The data presented in this study are available on request from the corresponding author. The data are not publicly available due to the privacy involved in the participants.

## Supplementary Materials

The following are available online at https://www.mdpi.com/1660-4601/18/4/1940/s1, Table S1: Introduction of interventions.
