# Supplementary material for: The Effectiveness of an Educational Intervention on Knowledge, Attitudes and Reported Practices on Antibiotic Use in Humans and Pigs: A Quasi-Experimental Study in Twelve Villages in Shandong Province, China
Source: Int J Environ Res Public Health. 2021 Feb 17;18(4):1940. doi: 10.3390/ijerph18041940 (PMC7922583; doi:10.3390/ijerph18041940)
Supplement: Supplementary file 1 [file ijerph-18-01940-s001.pdf]

Supplementary Materials

Table S1. Introduction of interventions.

Interventions	Distribution	Contents	Frequency
Training session	Two chief physicians from the county hospital	· Introduction to antibiotics;<br>· Consequences of antibiotic overuse<br>· The causes of antibiotic resistance;<br>· How to use antibiotics rationally;<br>· How to best deal with the common cold and diarrhea in children.	Every season during the one-year intervention (4 times in total)
Speakerphone	Playback coordinated by a social worker in each village	· Basic knowledge of antibiotics;<br>· Rational antibiotic use for children;<br>· Rational antibiotic use for pigs	Two times (morning and evening) every Tuesday, Thursday and Saturday during the one-year intervention
Posters	Placed at key gathering points by the Research team	· Knowledge of upper respiratory tract infections and the immune system.<br>· Rational antibiotic use for humans;<br>· Potential for adverse drug reactions caused by antibiotics.	Replaced with new ones once a quarter
Handbook	Delivered to households by village doctors	· Definition of antibiotics;<br>· Antibiotic use indications;<br>· Harm of irrational antibiotic use for humans;<br>· How to use antibiotics rationally for humans.	Distributed after training session (4 times in total)
